# Endometrial and vaginal microbiome in donkeys with and without clinical endometritis

**DOI:** 10.3389/fmicb.2022.884574

**Published:** 2022-08-01

**Authors:** Jing Li, Yiping Zhu, Junpeng Mi, Yufei Zhao, Gilbert Reed Holyoak, Ziwen Yi, Rongzheng Wu, Zixuan Wang, Shenming Zeng

**Affiliations:** ^1^Equine Clinical Diagnostic Center, College of Veterinary Medicine, China Agricultural University, Beijing, China; ^2^National Engineering Laboratory for Animal Breeding, Key Laboratory of Animal Genetics and Breeding of the Ministry of Agriculture, College of Animal Science and Technology, China Agricultural University, Beijing, China; ^3^School of Veterinary Science, University of Sydney, Sydney, NSW, Australia; ^4^College of Veterinary Medicine, Oklahoma State University, Stillwater, OK, United States; ^5^College of Veterinary Medicine, Kansas State University, Manhattan, KS, United States

**Keywords:** donkeys, endometritis, reproductive tract, microbiome, high-throughput sequencing

## Abstract

Endometrial and vaginal microbiomes are critical in the study of endometritis, which is an important cause of infertility in donkeys. Our objective was to investigate the difference of the endometrial and vaginal microbiomes between healthy donkey jennies (group C) and jennies with endometritis (group E). Endometrial and vaginal swab samples were collected, and the 16 s rRNA gene amplicon high-throughput sequencing technique was applied to identify the microbial composition in the samples. A similar microbial composition pattern was found between endometrial and vaginal samples, which indicated the impact of the vaginal microbiome on the endometrial microbial environment and health. There was a significant difference of endometrial and vaginal swab samples between the two groups. *Ruminococcaceae* and *Lachnospiraceae* were significantly more abundant in endometrial and vaginal microbiomes of group E than in group C. Their dominance was consistent with increased anaerobic bacterial taxa in the functional analysis, which might be associated with the pathogenesis of endometritis in donkeys. *Sphingomonadaceae*, a bacterial family reported in bovine semen, was statistically more abundant in endometrial microbiome of group E than in group C, which might suggest an association between high abundance of *Sphingomonadaceae* possibly due to uncleared semen and donkey endometritis. Our study revealed the composition of the vaginal and endometrial microbiomes in healthy and endometritis donkeys. These findings will provide more insights into the pathogenesis of donkey endometritis.

## Introduction

Endometritis is an inflammation of the endometrial layer and is considered the main cause of infertility in mares (Troedsson, 1999). Traditionally, endometritis has been divided into infectious or noninfectious causes. Infectious endometritis in mares includes acute and chronic infections, while noninfectious endometritis is commonly referred to as post-mating-induced endometritis (PMIE; LeBlanc, 2010). Equine endometritis, in most cases, is initiated by the normal physiological inflammatory responses to breeding involving a two-pronged mechanical and immunological response to clear the uterus of semen and bacteria. If that fails, a persistent breeding-induced endometritis (PBIE) occurs (Morris et al., 2020). Occasionally, endometritis can also develop subsequent to hematogenous infections (Agerholm, 2013; Rantala et al., 2015). Individuals that fail to clear inflammatory insults, including infectious or noninfectious agents, will be more prone to infectious endometritis (LeBlanc and Causey, 2009). Routinely, culture-based methods have been used to study the microbial composition of the equine uterus with endometritis (LeBlanc and Causey, 2009). However, 16 s rRNA gene amplicon high-throughput sequencing technique has been applied in humans (Moreno and Simon, 2018) and other mammals such as canine (Lyman et al., 2019) and bovine (Wang et al., 2018) to explore the broader uterine microbiome, which provided better understanding of the microbial richness and diversity within the uterus. In dairy cattle, *Fusobacterium*, *Trueperella*, and *Peptoniphilus* were demonstrated to be associated with clinical endometritis (LeBlanc and Causey, 2009). The healthy equine uterine microbiome has recently been investigated, and distinct composition was found compared with other species (Holyoak et al., 2018; Jones, 2019). To our knowledge, to date, there have been no published reports on the microbiome composition of equine endometritis.

The vaginal vestibule is one of the important anatomical barriers for uterine defense (Barba et al., 2020). There is evidence that in humans, the normal vaginal microbiome can create an acidic environment due to the dominance of *Lactobacillus* to protect women’s reproductive tract against bacterial ascension (Miller et al., 2016). Even though *Lactobacillus* has been found much less abundant in mares, the vaginal microbiome is still considered critical to the health of the vaginal environment and reproductive efficacy (Mahalingam et al., 2019). Additionally, the vaginal microbiome of infertile women has been compared with that of healthy women, and specific microorganisms were identified as potential biomarkers to help diagnose infertility (Campisciano et al., 2017). The normal vaginal microbiome in horses has recently also been investigated, and opportunistic microorganisms such as *Streptococcus* spp. and *Enterobacteriaceae* spp. were detected in healthy mares (Jones, 2019; Husso et al., 2020). It has also been found that the ovarian cycle does not affect the composition of the vaginal microbiome in Arabian horses (Barba et al., 2020).

Due to the proximity of the uterus to the vagina, vaginal bacteria may inadvertently seed into uterus during placement of intrauterine devices (Mitchell et al., 2015). Therefore, microbiome studies of the vagina and uterus could help elucidate the microbial relationship between the two compartments. A comparative study of the vaginal microbiome and the uterine microbiome has been conducted on women with chronic endometritis (Lozano et al., 2021). The result demonstrated characteristic vaginal and uterine microbiomes, as well as a clear association between the vaginal microbiome and chronic endometritis. In dairy cows, comparative evidence showed that there was a mixing of vaginal and uterine microbiomes in cases of postpartum endometritis (Miranda-CasoLuengo et al., 2019). This has been known for many years through culture-based investigations (Elliott et al., 1968; Sheldon et al., 2006).

Donkey endometritis has been an issue affecting donkey fertility in the Chinese donkey industry (Gao et al., 2020). However, studies are scarce on donkey endometritis around the world (Rota et al., 2012; Gao et al., 2020). To our knowledge, donkey endometrial and vaginal microbiomes have not been previously investigated. In the current study, we applied the 16 s rRNA gene amplicon high-throughput sequencing technique for the first time and compared the endometrial and vaginal microbiomes of swab samples between healthy donkey jennies (group C) and donkey jennies with endometritis (group E). Our objective was to characterize the normal endometrial and vaginal microbiomes in donkeys and illustrate the impact of donkey endometritis on the endometrial and vaginal biological environments.

## Materials and methods

All animal procedures were conducted after obtaining approval from the Animal Care and Use Committee of the China Agricultural University (No. AW51211202-2-1).

### Experimental design and sample collection

This study was performed in a donkey farm located in Hebei Province, China. There were about 500 Yangyuan donkeys in total raised on that farm, and 80% of them were breeding donkey jennies (*Equus asinus*). The donkeys were all fed hay and homemade corn concentrates, with free access to water daily. A total of 22 donkey jennies were enrolled in this study. All jennies were multiparous, ranging from 4 to 8 years of age. During Sep 2020 to Nov 2020, 10 jennies were enrolled in the study with a history of conception failure after artificial insemination, vaginal discharge, and increased intrauterine fluid accumulation 48 h after insemination detected by ultrasonographic examination (Morris et al., 2020). These donkeys were presumably diagnosed to have clinical endometritis based on the results of the breeding history (Supplementary Table 1), physical examination, and transrectal ultrasonography (LeBlanc and Causey, 2009). They were assigned to group E, and their endometritis would be confirmed by further endometrial cytology and culture. Totally, 12 healthy donkey jennies with normal reproductive histories (Supplementary Table 2) and no clinical signs were recruited as controls and assigned to group C.

Endometrial microbial samples were collected with sterile double-guarded swabs (IMV Technologies, L’Aigle, France) before any treatment. The tail was wrapped with a brown gauze before all procedures. The vulva and perineum were scrubbed with soap and water to minimize contamination (Albihn et al., 2003). Sterile obstetrical sleeves were applied to protect and guide the double-guarded swabs through the cervix. A sterile obstetrical lubricant was used to lubricate the sleeve before entering the reproductive tract. The swab was extended from the protective tube to swab the surrounding endometrium for 10–15 s and then drawn back into the tube before withdrawing from the uterus. Finally, three separate swabs were collected from each donkey for compositional analysis of the microbiota, bacterial culture, and cytology, respectively. The swabs for the microbiome samples were collected first and then bacterial culture and cytology samples were collected. The endometrial swab sample for aerobic culture (Katila, 2016) from each donkey jenny was soaked with 1 ml saline to dilute and mobilize the bacteria. All swab samples were stored on ice temporarily for no more than 2 h before arriving at the laboratory. The swabs for culture and cytology were processed within 2 h after sampling. The swabs for the microbiome were stored at −80°C until further analysis. The samples collected from group C were collected with the same methods as described before.

Vaginal samples were also collected from all jennies with the same type of double-guarded swab as used for the endometrium. After sampling from the uterus, a sterile obstetrical sleeve was applied to protect and guide the protective tube through the vestibule to the vagina. Vaginal samples were collected from the mucosal epithelium of the mid-vaginal floor, using the same basic procedure was performed in the uterus. Only one vaginal swab was collected from each jenny for microbiome analysis. The vaginal swabs were stored at −80°C upon arrival at the laboratory until further analysis.

Totally, three negative control samples for microbiota were collected with the same supply of sterile sleeves and lubricant that were used in the endometrial and vaginal sample collection. The swab was protected by the sleeve in the same way described earlier. It was then protruded and drawn back without going into the reproductive tract. The negative control swab samples were also processed in the same way and likewise stored until further analysis.

### Aerobic culture and cytology

After arriving at the laboratory, two of the endometrial swabs collected from each member of group E and group C were processed for culture and cytology, respectively. The swab samples soaked in saline were then inoculated on Columbia blood agar (Huankai Microbial, Guangzhou, China) and MacConkey agar using an inoculation loop for bacterial culture at 37°C. Each agar plate was inspected for bacterial growth after 24- and 48-h inoculation. Colonies were counted and graded at 48 h following established criteria (Christoffersen et al., 2012): no growth/sterile, <5 CFU/per plate; mild growth, 5–10 CFU/plate; moderate growth, 11–50 CFU/plate; and heavy growth, >50 CFU/plate. The growth of more than three different types of bacterial colonies was recorded as contamination. Further bacterial identifications were conducted by matrix-assisted laser desorption ionization-time-of-light mass spectrometry (MALDI-TOF MS).

The other swab was rolled on a microscope slide for cytology evaluation. The cytology slides were stained using the Diff-Quik^R^ system (Baso Biotechnology Co., Ltd., Zhuhai, China). The evaluation of cytology slides was performed under a light microscope (400 × magnification) for the presence of neutrophils per high-power field (hpf). Samples with a number of neutrophils ≥2 per hpf over 10 fields with endometrial cells present were considered positive for endometritis (Riddle et al., 2007).

Jennies presumably diagnosed with endometritis would be confirmed if their endometrial swab samples had both a mild or higher grade of bacterial growth and positive cytology results. Jennies in group C would be considered free of endometritis if their endometrial samples had <5 CFU/per plate and negative cytology results.

### DNA extraction and 16S rRNA gene amplicon sequencing

The swabs of all vaginal and endometrial samples as well as negative control samples were thawed from −80°C to room temperature in a controlled environment for DNA extraction using a PowerSoil^®^ DNA Isolation Kit (Qiagen, Hilden, Germany) according to manufacturer’s instructions. Purity and concentration of DNA products were determined by using a spectrophotometer (NanoDrop 2000, Thermo Scientific, Wilmington, United States; Supplementary Table 3). A two-step PCR protocol was carried out for sequencing library preparation. In the first step, PCR was conducted to amplify the genomic DNA of all samples using a primer set of 338F (5′- ACTCCTACGGGAGGCAGCA-3′) and 806R (5′- GGACTACHVGGGTWTCTAAT-3′) targeting the bacterial V3-V4 hypervariable region. The PCR was carried with 30 μl volume comprising 0.3 μM 338F, 0.3 μM 806R, 0.6 μl KOD FX Neo enzyme, 15 μl KOD FX Neo buffer (TOYOBO, Shanghai, China), and 0.4 mM dNTP. The PCR was conducted at 98°C for 2 min at the denaturation phase, followed by 30 thermal cycles (98°C, 30 s for denaturing, 50°C, 30s for annealing and 72°C, 5 min for extension) for amplification and a single extension at 72°C for 5 min. In the second round of PCR, dual-indexed sequences and adaptor sequences were added with 10 cycles in a 20 μl reaction volume. Successful DNA amplification products were verified by 1.8% agarose gel electrophoresis and purified using the E.Z.N.A Gel Extraction kit (Omega Bio-Tek, Norcross, United States). Purified amplicons were pooled and paired-end sequenced on an Illumina Novaseq 6,000 PE 250 system (Illumina, San Diego, United States).

### Bioinformatics analysis

Raw sequencing data in the form of FASTQ files were analyzed using QIIME2 (Bolyen et al., 2019). Data demultiplex and quality filter were also performed on this bioinformatics pipeline. Reads were truncated, with an average quality score < 20 in a 50-bp window. Operational taxonomic units (OTUs) below an abundance of 0.005% were filtered. OTUs were clustered with 97% similarity cutoff using UPARSE (version 7.1; Edgar, 2013). Chimeric sequences were also identified and removed with the Uchime algorithm (version 4.2.40; Edgar et al., 2011). RDP classifier (v 2.2) was applied to analyze the taxonomy of each OTU representative sequence using the Silva (v138) database, with a minimum threshold of 0.7.

### Statistical analysis

The OTUs reaching a 97% similarity level were analyzed in QIIME 2. Alpha diversity was analyzed using Shannon (diversity) and Chao (richness) indexes. Student’s *t*-test was performed to illustrate the differences of the indexes between the two groups. Differences in community composition were demonstrated by analysis of variance (ANOVA) and the Mann–Whitney U test with a normalized relative abundance matrix. Beta diversity analysis was assessed by the principal coordinate analysis (PCoA) based on the Bray–Curtis distance. Relative abundance of taxa at different levels were summarized in bar plots. Linear discriminant analysis (LDA) effect size (LEfSe; Segata et al., 2011) was also applied with an LDA score of >4 to identify the differentiated bacteria between group C and group E in the endometrium and vagina, respectively. Functional analysis was conducted with BugBase (Ward et al., 2017) and functional annotation of prokaryotic taxa (FAPROTAX) on QIIME2. OTUs were categorized into different classes including aerobic, anaerobic, and facultatively anaerobic on BugBase website. OTUs were annotated, and bacterial genes were predicted for potential functional traits by using the FAPROTAX database (Jeon et al., 2015).

### Quantitative PCR

Of the extracted DNA products from the donkey swab samples in the previous step, four were amplified in a 20-μl PCR system including 0.5 μM 338F, 0.5 μM 806R, 10 μl Premix LA Taq^TM^ (Takara Bio USA Inc. San Jose, United States), and 1 μl DNA product. The reaction was conducted at 95°C for 5 min at the denaturation phase, followed by 30 thermal cycles (95°C, 30 s for denaturing, 50°C, 30 s for annealing and 72°C, 30 s for extension) for amplification and a single extension at 72°C for 10 min. The electrophoresis-verified and gel-purified PCR products were TA cloned using a pClone007 Versatile Simple Vector Kit (007 *VS*; Tsingke Biotechnology Co., Ltd., Wuhan, China) following the manufacturer’s instructions. Plasmid isolation was achieved using the Plasmid DNA Miniprep Kit (PM0201-200; Tsingke Biotechnology Co., Ltd., Wuhan, China). The purity and concentration of DNA were detected by using a NanoDrop 2000 Spectrophotometer (Thermo Scientific, Waltham, United States). Confirmation of the cloned 16S rRNA gene was performed by sequencing four of the isolated plasmids. The plasmid clone with a sequence that shared greatest identity (100%) with a published 16S rRNA gene (GenBank accession # KF089531.1) was selected as the DNA standard. The 10-fold serial dilutions (10^6^–10 copies/ml) of the DNA standard were prepared with three replicates of each dilution. The quantification of standard DNA was performed, and the threshold cycle (C_t_) of the plasmid genome dilutions was plotted against the log number of copies to construct the standard curve.

The qPCR of all samples, including negative controls, contained 1 μl 10-fold-diluted extracted DNA product, 0.5 μM 338F, 0.5 μM 806R, and 10 μl 2 × T5 Fast qPCR Mix (SYBR Green I; Tsingke Biotechnology Co., Ltd., Wuhan, China). A total qPCR system of 20 μl was achieved with the addition of double distilled water. qPCR assays were performed with a thermo cycling profile comprising 2-min initial denaturation at 95,°C followed by 40 cycles of 95°C for 15 s, 56°C for 20 s, and 72°C for 20 s with fluorescence data collection during the 72°C step. Then three replicates of each sample genome including negative controls were run to determine the mean and standard deviation of copy numbers for each sample genome. The data of C_t_ values obtained in qPCR assays were assembled in Microsoft Excel. The log numbers of genome copies were calculated based on the standard curve.

## Results

### Aerobic culture and cytology results

All donkeys from group E were positive for culture and cytology from the collected endometrial samples. Non-hemolytic *Escherichia coli* (*E. coli*) was the dominant bacterium (>90% of the grown colonies per plate) cultured from all endometrial swabs from group E, which was considered to have substantial growth in monoculture (Nielsen, 2005). Therefore, *E. coli* was recorded in the culture results of group E (Table 1). These jennies also had >2 neutrophils per hpf. All plates grew less than three types of bacterial colonies. Therefore, no plates were recorded to have contamination in this study. Based on the culture and cytology results, donkeys in group E were diagnosed as having endometritis. Jennies from group C were all negative for the endometrial cytologic examination. There were few bacterial colonies (<5 CFU/per plate) noted in the endometrial swab cultures from group C jennies. According to the criteria applied in this study, the culture was classified as no growth. Therefore, donkey jennies in group C were considered free of endometritis at this point.

**Table 1:** Bacterial culture and cytology results from uterine swab samples from 22 donkey jennies.

	Sample	Group	Colony counts/CFU	Bacterial culture results	Cytology/hpf^b^
1	Z2649	control	<5	ng^a^	0–1 PMN^c^
2	Z1638	control	<5	ng	0–1 PMN
3	Z1914	control	<5	ng	0–1 PMN
4	Z1888	control	<5	ng	0–1 PMN
5	Z0705	control	<5	ng	0–1 PMN
6	Z0591	control	<5	ng	0–1 PMN
7	Z000	control	<5	ng	0–1 PMN
8	Z1650	control	<5	ng	0–1 PMN
9	Z180	control	<5	ng	0–1 PMN
10	Z1079	control	<5	ng	0–1 PMN
11	Z1917	control	<5	ng	0–1 PMN
12	Z314	control	<5	ng	0–1 PMN
13	Z2815	control	<5	ng	0–1 PMN
14	ZN0123	endometritis	5–10	E.coli, mild	2–5 PMNs
15	ZN1489	endometritis	5–10	E.coli, mild	2–5 PMN
16	ZN1781	endometritis	5–10	E.coli, mild	5–10 PMN
17	ZN2794	endometritis	5–10	E.coli, mild	2–5 PMNs
18	ZN2712	endometritis	11–50	E.coli, moderate	2–5 PMNs
19	ZN1296	endometritis	11–50	E.coli, moderate	2–5 PMN
20	ZN0250	endometritis	11–50	E.coli, moderate	2–5 PMNs
21	ZN1587	endometritis	>50	E.coli, severe	10–15 PMNs
22	ZN1057	endometritis	>50	E.coli, severe	5–10 PMNs
23	ZN0115	endometritis	>50	E.coli, severe	5–10 PMNs
24	ZN2918	endometritis	>50	E.coli, severe	>20 PMNs

a ng, negative growth;

b hpf, high-power field;

c PMN, polymorphonuclear leukocytes.

### Sequencing data overview

**Figure 1:**
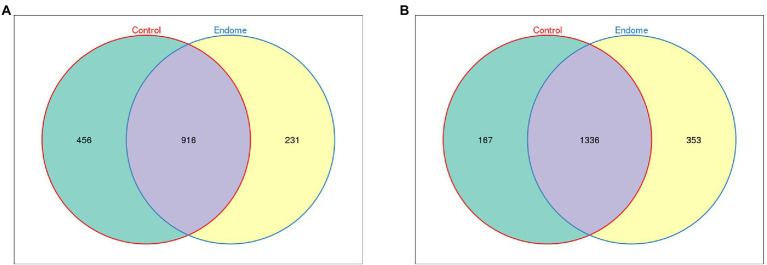
Venn diagram demonstrating the exclusive and common OTUs of group E and group C. **(A)** Endometrial microbiota of group E and group C. **(B)** Vaginal microbiota of group E and group C.

There was no target (16 s rRNA V3-V4 region) DNA product yielded from negative control samples after the two-step PCR protocol. Hence, it was concluded that the amount of bacterial DNA in negative control swabs was negligible (Siddiqui et al., 2011). Endometrial and vaginal swabs taken from group E and group C were evaluated by the 16S rRNA gene amplicon high-throughput sequencing technique. Rarefaction curves (Supplementary Figures 1A,B) were performed between group E and group C in the endometrial and vaginal microbiota, respectively, and the data showed sufficient sequencing depth to cover the overall bacterial diversity. Most sample libraries yielded about 80,000 clean reads (Supplementary Table 3), and all libraries were normalized to the smallest size of library (61,501 reads) for further data analysis. Totally, 1,735,530 sequence reads and 1,603 OTUs in total were identified in all endometrial swab samples; 1,372 and 1,149 OTUs were from group C and group E, respectively, while 916 OTUs were shared by the two groups (Figure 1A). In the vaginal swab samples, a total of 1,913,005 sequence reads and 1856 OTUs were detected. The Venn diagram showed that 1,503 and 1,689 OTUs in total were identified from group C and group E, respectively, with 1,336 shared by the two groups (Figure 1B). Raw sequence data of all samples were deposited at Sequence Read Archive (SRA) database of the NCBI (accession number PRJNA788693).

### Bacterial sequencing analysis of the endometrial microbiome

**Figure 2:**
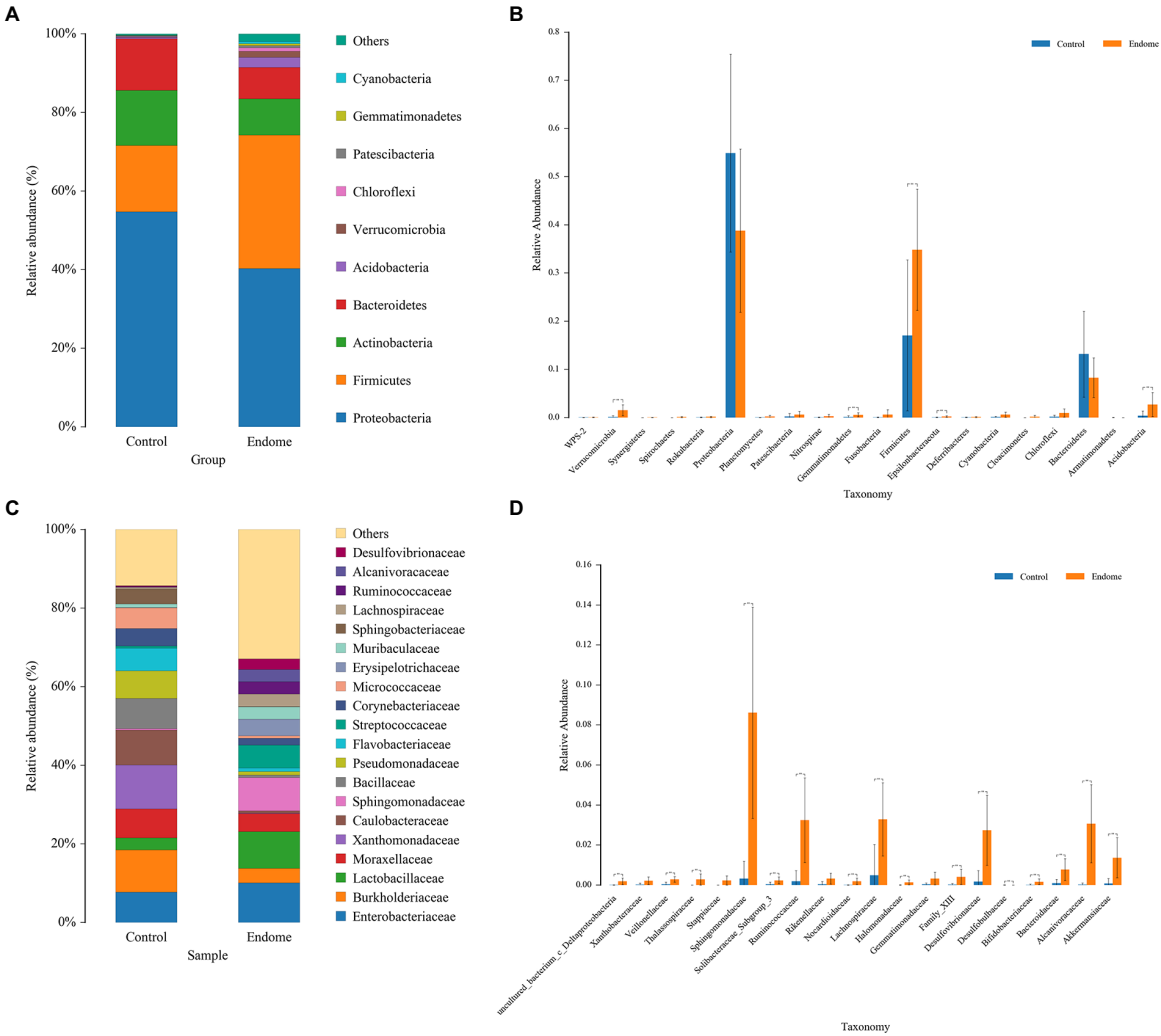
Bacterial taxon sequencing analysis of endometrial microbiota in donkey jennies of group C and group E. **(A)** Relative abundance of OTUs at the phylum level of the two groups. **(B)** ANOVA of endometrial microbiota at the phylum level between group E and group C. Blue bars represent group C, and orange bars represent group E. ^**^Significant difference (*p* < 0.01; Mann–Whitney U test). **(C)** Relative abundance of OTUs at the family level of the two groups. **(D)** ANOVA of endometrial microbiota at the family level between group E and group C. Blue bars represent group C, and orange bars represent group E. ^**^Significant difference (*P* < 0.01; Mann–Whitney U test).

In investigating the differences in the microbiome in samples from the endometrium and the vagina of group C and group E, Proteobacteria, Firmicutes (now Bacillota), and Actinobacteria were the most abundant phyla in the endometrial microbiomes of both groups (Figure 2A). Other phyla with lower relative abundance included Bacteroidetes, Acidobacteria, Verrucomicrobia, and Chloroflexi. Only for the phylum Firmicutes, there was a significant difference (*p* < 0.01) between the two groups (Figure 2B). At the family level, *Enterobacteriaceae*, *Burkholderiaceae*, and *Lactobacillaceae* were the relatively dominant bacterial taxa in endometrial samples (Figure 2C). *Sphingomonadaceae*, *Ruminococcaceae*, *Lachnospiraceae*, and *Desulfovibrionaceae* in group E had significantly higher relative abundance (*p* < 0.01) than group C (Figure 2D).

**Figure 3:**
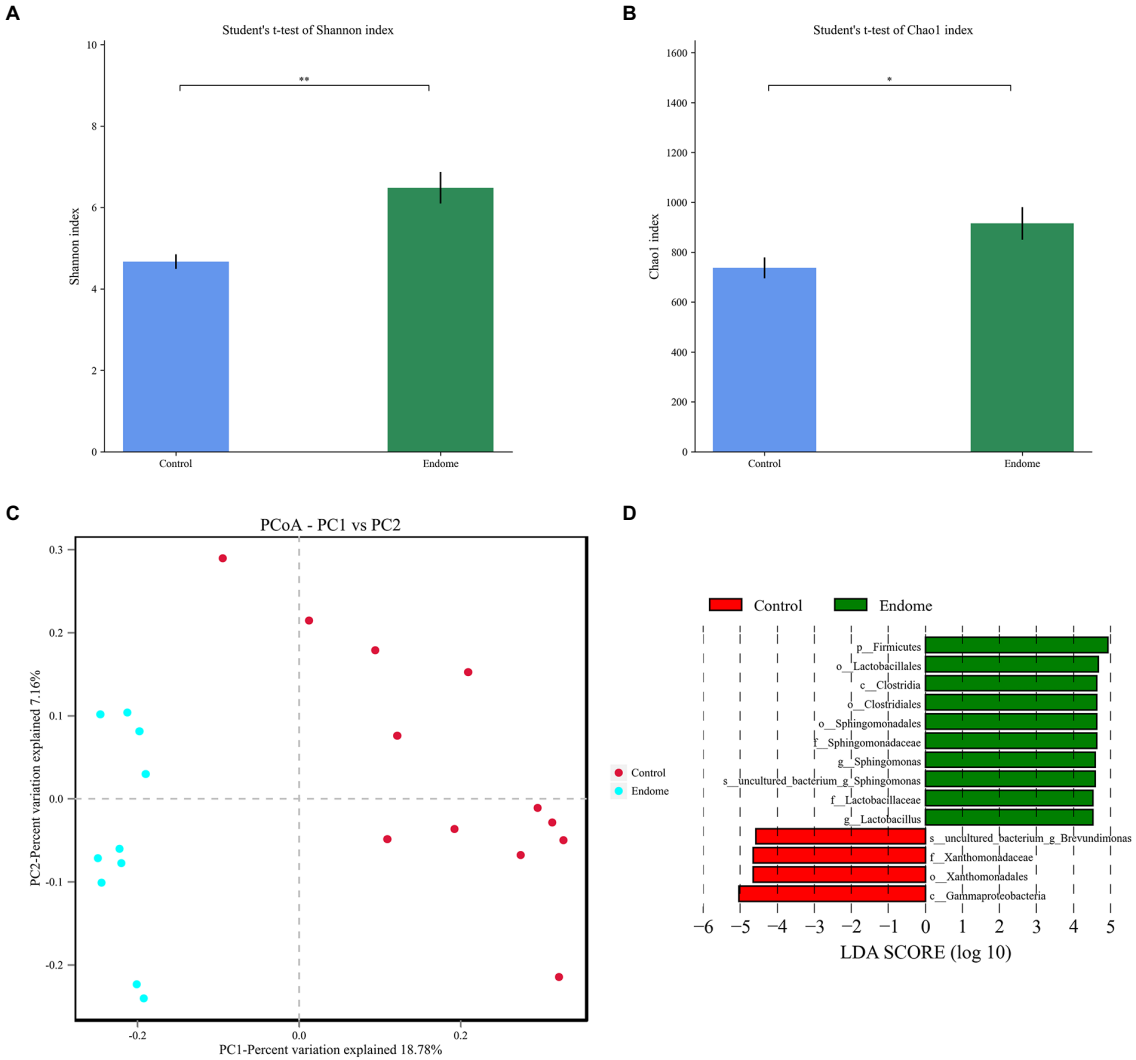
Bacterial diversity analysis of endometrial microbiota in donkey jennies of group C and group E. Shannon index **(A)** and Chao index **(B)** of the OTU level in endometrial microbiota of group C and group E. C: Healthy donkeys jennies. E: Donkey jennies with endometritis. ^*^Significant difference (*P* < 0.05, Student’s *t*-test). ^**^Significant difference (*p* < 0.01, Student’s *t*-test). **(C)** Principal coordinate analysis (PCoA) of the endometrial microbiota of healthy and endometritis donkeys based on Bray–Curtis distance. C: Healthy donkeys (*n* = 12) in red; E: endometritis donkeys (*n* = 10) in blue. **(D)** Linear discriminant effect size analysis (LEfSe) of the endometrial microbiota of healthy and endometritis donkeys. Bacterial taxa at the genus level and higher in group C (healthy donkeys, in red) and group E (endometritis donkeys, in green) were illustrated by LDA scores >4.

In terms of alpha diversity in the endometrial sample microbiomes, there were significant differences in the Shannon index, which provides information about both richness and evenness in the microbiome (*p* < 0.01; Figure 3A), and Chao index, which estimates total richness (*p* < 0.05; Figure 3B) between the two groups. The Shannon index and Chao index of group E were both statistically higher than those in group C (*p* < 0.05). The beta diversity analysis, which is a measure of similarity or dissimilarity of the two microbiome communities, and principal coordinate analysis (PCoA), based on the Bray-Curtis distance, illustrated two distinct clusters representing the endometrial microbiome samples of groups E and C (Figure 3C).

LEfSe with an LDA score > 4 was demonstrated to identify the bacterial taxa associated with endometritis (Figure 3D). Compared to group C donkeys, Sphingomonadales (order), *Sphingomonadaceae* (family), and *Sphingomonas* (genus) had higher LDA scores in group E, indicating a stronger association with endometritis over the control animals.

### Bacterial sequencing analysis of the vaginal microbiome

**Figure 4:**
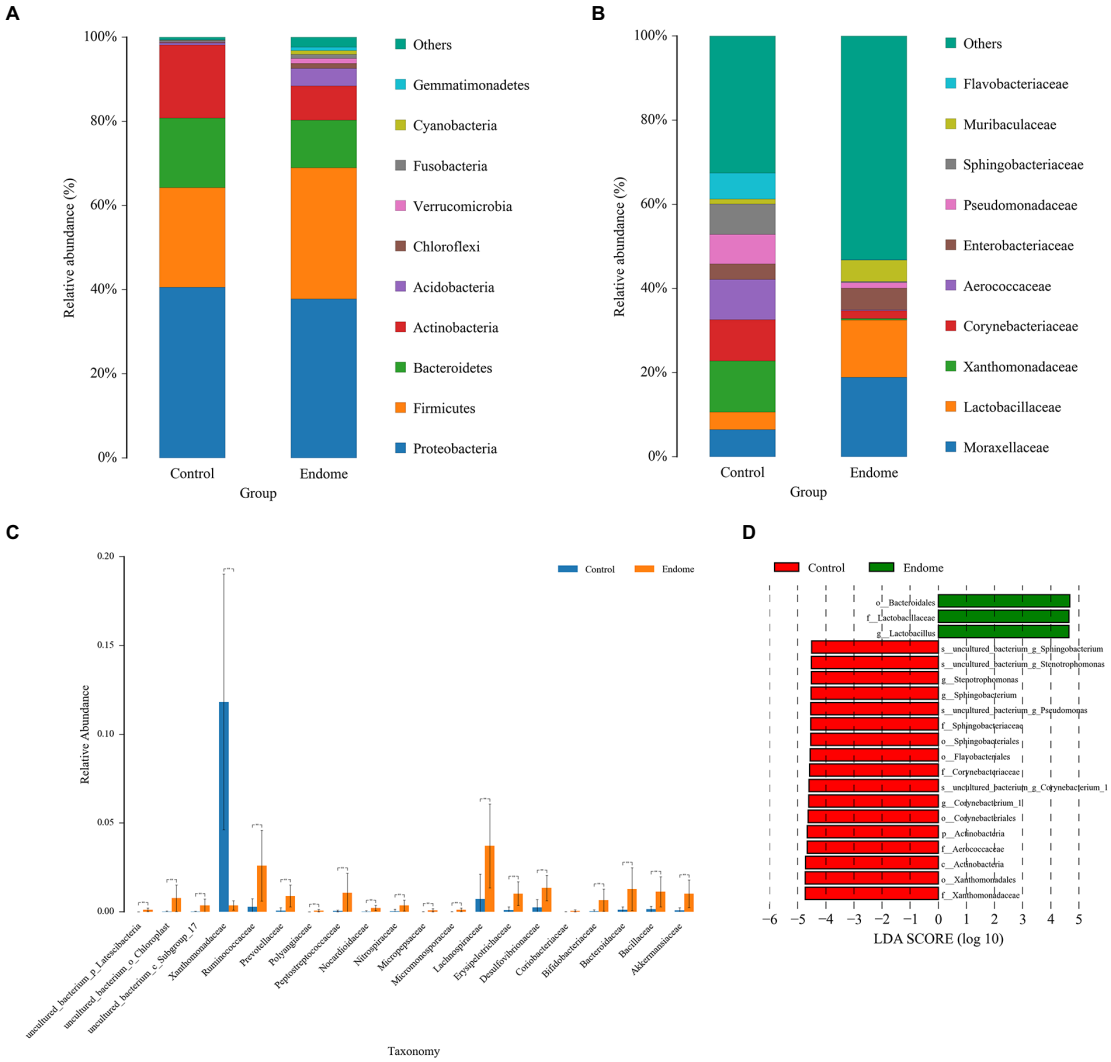
Bacterial taxon sequencing analysis of vaginal microbiota in donkey jennies of group C and group E. **(A)** Relative abundance of OTUs at the phylum level of the two groups. **(B)** Relative abundance of OTUs at the family level of the two groups. **(C)** ANOVA of vaginal microbiota at the family level between group E and group C. Blue bars represent group C, and orange bars represent group E. ^**^Significant difference (*p* < 0.01; Mann–Whitney U test). **(D)** Linear discriminant effect size analysis (LEfSe) of the vaginal microbiota of healthy and endometritis donkeys. Bacterial taxa at the genus level and higher in group C (healthy donkeys, in red) and group E (endometritis donkeys, in green) were illustrated by LDA scores > 4.

Proteobacteria, Firmicutes, and Bacteroidetes had the highest abundance in the vaginal microbiome of both group E and group C (Figure 4A). Actinobacteria, Acidobacteria, Chloroflexi, and Verrucomicrobia with lower relative abundance were also present in the endometrial microbiota in this study. At the family level, the relative abundance distribution of *Lactobacillaceae*, *Xanthomonadaceae*, and *Enterobacteriaceae* in vaginal group C and group E displayed a similar pattern to their distribution in endometrial group C and group E (Figure 4B). *Aerococcaceae* was predominantly identified in vaginal samples, especially from healthy donkeys (Figure 4B). The significantly different bacterial families between the two groups included *Xanthomonadaceae* (*p* < 0.001), *Ruminococcaceae* (*p* < 0.001), and *Lachnospiraceae* (*p* < 0.001; Figure 4C). The PCoA demonstrated a clear separation of the vaginal microbiome samples from group C and group E.

The bacterial taxa in the vaginal microbiome from group E associated with endometritis were demonstrated by LEfSe with an LDA score > 4. The bacterial taxa in vaginal microbiome most associated with endometritis include *Lactobacillaceae* (family) and *Lactobacillus* (genus; Figure 4D).

### Functional analysis of endometrial and vaginal microbiome

**Figure 5:**
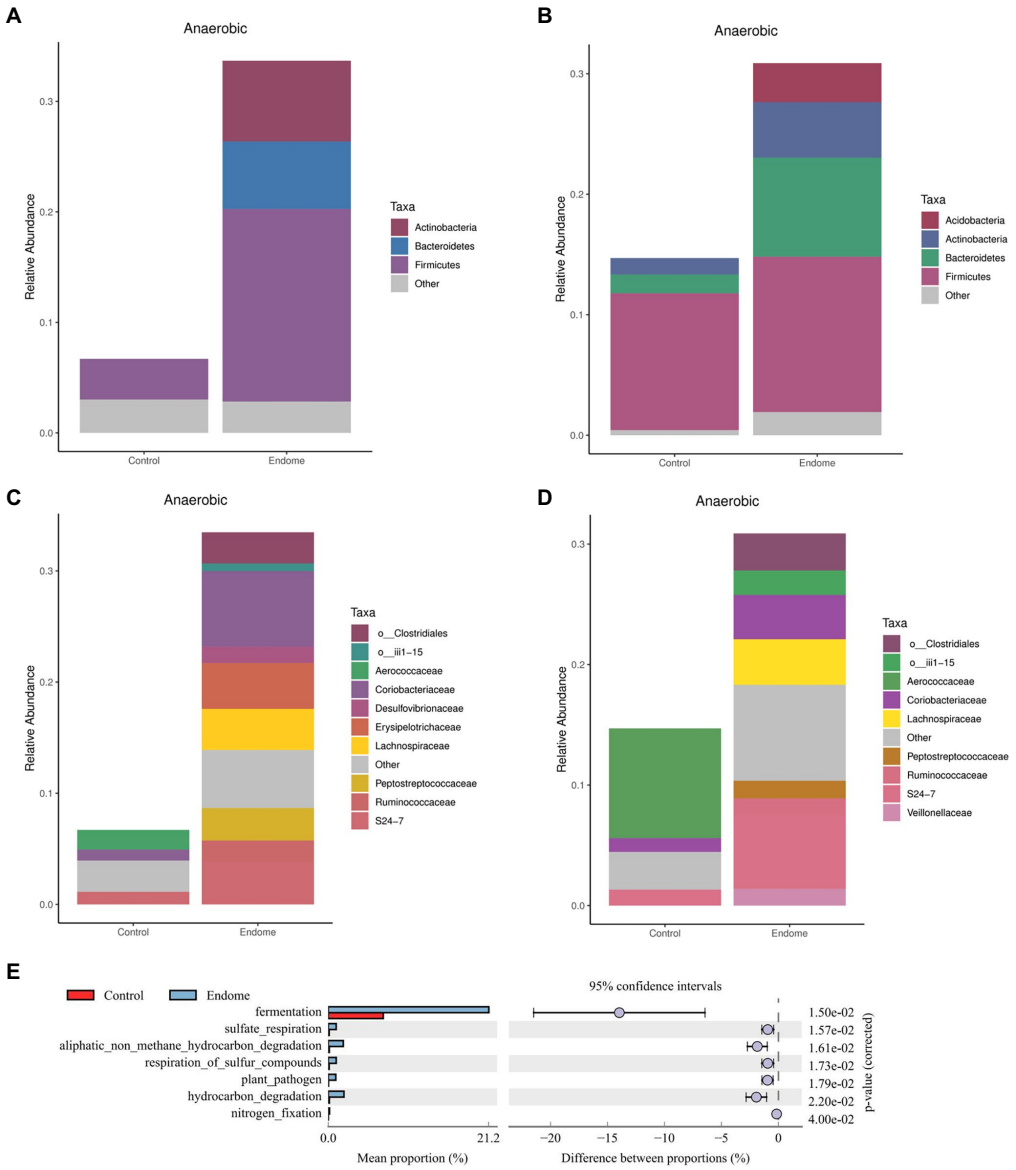
Functional analysis of bacterial taxa in healthy donkeys (group C) and endometritis donkeys (group E). Relative abundance of anaerobic bacterial taxa at the phylum level in endometrial **(A)** and vaginal **(B)** microbiota of group C and group E based on BugBase analysis. Relative abundance of anaerobic bacterial taxa at the family level in endometrial **(C)** and vaginal **(D)** microbiota of group C and group E based on BugBase analysis. **(E)** Functional genes were predicted in uterine community of the two groups illustrated by the extended error bar plot of abundance profiles at class 1. Blue represents group E, and red represents group C.

There was a dramatically increased relative abundance of anaerobic bacterial taxa in both the endometrial and vaginal microbiomes of donkeys with endometritis at the phylum and family levels. In the endometrial microbiome, Firmicutes, Bacteroidetes, and Actinobacteria were the main phyla involving the anaerobic bacterial taxa (Figure 5A). In the vaginal microbiome, changes in the anaerobic bacteria statistically occurred in Bacteroidetes, Actinobacteria, and Acidobacteria (Figure 5B). At the family level, the notably increased anaerobic bacterial taxa in endometrial (Figure 5C) and vaginal (Figure 5D) microbiomes included *Ruminococcaceae*, *Peptostreptococcaceae*, and *Lachnospiraceae*.

An extended error bar plot of abundance profiles at class 1 (Figure 5E) depicted the endometrial community composition for predicted functional genes. Fermentation-related genes differed most significantly (corrected value of *p* = 0.015) between healthy jennies and jennies with endometritis.

### Data analysis of qPCR assays

Linear regression of the standard curve was conducted to obtain the slope (Supplementary Figure 2). The qPCR assay for standard curve construction had a reaction efficiency of *E* = 85.2% (*r^2^* = 0.998). The log number of genome copies of all samples and negative controls were calculated and converted to the copy numbers. The average copy number of each sample, including negative controls, were recorded (Supplementary Table 4).

## Discussion

This is the first study utilizing the 16 s rRNA gene amplicon high-throughput sequencing technique to reveal the microbial composition of the endometrium and vagina in healthy donkeys, as well as donkeys with endometritis. We also detected significant differences within the endometrial and vaginal microbiota between donkey jennies with and without endometritis. The compositional difference of endometrial and vaginal microbiota between jennies with endometritis and healthy jennies indicate a significant impact of endometritis on the microbiota of the reproductive tract in donkey jennies. The bacterial richness and diversity were significantly higher in endometrial samples from jennies with endometritis than in those of healthy jennies. These results indicate that endometritis might lead to a disturbance in normal bacterial taxa, which play an important role in sustaining the health of the reproductive tract as described by Wang et al. (2016) in cattle. Although the impact of enhanced diversity and richness of endometrial microbiota on donkey jennies with endometritis is currently unknown, this variability probably induces an immune response, which contributes to disease pathophysiology.

Microbial compositions of vaginal and endometrial microbiomes of donkey jennies were demonstrated in the present study. In jennies involved in this study, the dominant phyla of endometrial and vaginal microbiomes were Proteobacteria, Firmicutes, and Actinobacteria. The uterine and vaginal microbiome of healthy mares were recently investigated (Holyoak et al., 2018; Jones, 2019; Husso et al., 2020). Bacteroidetes, Actinobacteria, and Firmicutes were the most abundant phyla of the normal uterine and vaginal microbiome in mares, even though there were differences of the relative abundance of each phyla between the two compartments (Jones, 2019). In cows, the most dominant bacterial phyla in the uterus and vagina were Firmicutes and Bacteroidetes (Miranda-CasoLuengo et al., 2019). By comparing the results of the present study with those of previous publications, the relative abundance distribution of those main phyla (Firmicutes, Proteobacteria, Bacteroidetes, and Actinobacteria) in donkey jennies’ reproductive tract was different from the distribution in the reproductive tract of cows and mares.

In the current study, we compared the vaginal microbiota with the endometrial microbiota to facilitate understanding of the influence of endometritis on the health of the jennies’ reproductive tract. The similar pattern of bacterial taxon distribution detected in the endometrial and vaginal microbiomes at both phylum and family levels in healthy jennies herein supports the theory that the colonization of uterine bacteria might come from the vagina, which has been found in other species as well (Moreno and Franasiak, 2017; Wang et al., 2017). In addition to the similarity of microbiota between the endometrium and vagina, characteristic bacterial taxa were also identified in each compartment. *Burkholderiaceae* is a bacterial family detected predominantly in endometrial samples of jennies, and it has also been reported with greater abundance in the uterine samples than in the vaginal samples of dairy cows (Lietaer et al., 2021). *Aerococcaceae* was identified as one of the dominant bacterial taxa in vaginal samples of jennies, and previously, it was also detected in vaginal samples of healthy gilts (Sanglard et al., 2020). The distribution difference of these characteristic bacterial taxa might need more attention to study the pathogenesis of donkey endometritis.

According to our results, Firmicutes was demonstrated as the only bacterial taxon at the phylum level of both the endometrial and vaginal microbiomes that displayed significantly higher relative abundance in jennies with endometritis than in healthy jennies. In sows and dairy cows, Firmicutes, the most dominant bacterial phylum in uterine microbiome, was found notably less abundant in animals with endometritis than in healthy animals (Miranda-CasoLuengo et al., 2019; Pascottini et al., 2020). The different patterns might be due to the different microbial compositions of the uterus between species. Based on the functional analysis of OTUs in the present study, Firmicutes contributed the most to the increase in the relative abundance of anaerobic bacterial taxa in jennies with endometritis. Hence, the shift to an anaerobic environment of the endometrium might be associated with the pathogenesis of donkey endometritis. This result might also indicate some limitations of aerobic culture in diagnosing donkey endometritis. Other phyla detected in endometrial microbiota in this study including Bacteroidetes, Acidobacteria, and Verrucomicrobia have been reported in uterine microbiota of cows (Chen et al., 2020) and mares (Jones, 2019). Chloroflexi is reported in equine endometrial microbiota for the first time in this study, even though its role in endometrial microbiota is still unknown.

At the family level, *Ruminococcaceae* and *Lachnospiraceae* were detected to be significantly more abundant in both endometrial and vaginal samples of donkey jennies with endometritis than in samples of healthy jennies. They did not have high relative abundance, being 0.2 and 0.5%, respectively, in healthy jennies, while they were more abundant (3.2 and 3.3%) in jennies with endometritis. However, *Ruminococcaceae* and *Lachnospiraceae* are critical families in the equine gut microbiome and responsible for gut homeostasis (Garber et al., 2020; Zhu et al., 2021). There was evidence that the two families could be translocated to the vagina from feces (Jones, 2019), which might explain their presence in the reproductive tract of donkey jennies in this study. In healthy cattle, *Ruminococcaceae* and *Lachnospiraceae* are common bacterial families of the vaginal and uterine microbiome (Ault et al., 2019). Their levels are relatively stable in the microbial community of the cows’ reproductive tract (Knudsen et al., 2016). Therefore, the shift of the relative abundance of *Ruminococcaceae* and *Lachnospiraceae* could be associated with disease in the reproductive tract. In one study, *Ruminococcaceae* and *Lachnospiraceae* were found to be present at a significantly lower relative abundance in uterine flush samples of cattle with endometritis (Knudsen et al., 2016). Increased relative abundance of *Ruminococcaceae* and *Lachnospiraceae* was also reported in vaginal microbiomes of dairy cows with endometritis (Miranda-CasoLuengo et al., 2019). However, in the present study, *Ruminococcaceae* and *Lachnospiraceae* were significantly more abundant in both vaginal and endometrial samples in donkey jennies with endometritis, which might indicate a different pathogenesis of endometritis in donkeys from dairy cattle.

In the functional analysis, *Ruminococcaceae* and *Lachnospiraceae* were the two main anaerobic bacterial families identified in endometritis donkeys. They are capable of degrading and fermenting substrates in the gastrointestinal system (Biddle et al., 2013). In the sow, it has been shown that some intestinal bacteria might be associated with the onset of sow endometritis (Zhang et al., 2021). According to the results in the current study, we speculate that alterations of intestinal bacterial taxa such as *Ruminococcaceae* and *Lachnospiraceae* could be associated with the occurrence of donkey endometritis. Nonetheless, further investigation of the correlation between endometritis and these bacterial taxa in donkeys is warranted.

*Sphingomonadaceae* has been identified in both vaginal and endometrial samples in the present study; however, a significantly higher abundance of *Sphingomonadaceae* was detected in endometrial samples of endometritis jennies than in those of healthy jennies. Hence, we hypothesized that an increased relative abundance of *Sphingomonadaceae* might be associated with donkey endometritis. Herein, *Sphingomonas* was discriminately enriched (LDA score > 4) in endometrial samples of endometritis donkey jennies according to LEfSe analysis, which indicated the existence of association between *Sphingomonas* and endometritis in donkeys. The same method was applied in both endometrial and vaginal sample collection for all donkey jennies, but a significantly higher level only occurred in endometrial samples from endometritis donkey jennies. Therefore, the higher abundance of *Sphingomonadaceae* was possibly associated with pathogenesis of endometritis. *Sphingomonas* spp. has been widely identified in nature, especially in aquatic environments (Koskinen et al., 2000). So far, they have been reported in samples of bovine ejaculated semen (Kilburn et al., 2013), which might be contaminated by environmental sources. In the current study, the samples of endometritis donkey jennies were collected within 4 to 6 days post-insemination, at which time the semen might not be completely cleared out from the uterus of jennies with endometritis. Hence, the higher abundance of *Sphingomonadaceae* in endometrial samples of endometritis jennies could indicate that it was introduced by semen during the artificial insemination (AI) and might be associated with a risk of endometritis if predominantly present. *Sphingomonadaceae* was also identified in endometrial samples of healthy jennies but with much lower relative abundance. Its presence might originate from previous breeding, but it was mostly cleared by healthy jennies and did not impact the stability of their endometrial microbiota. Further study will be required to investigate the association between donkey semen and *Sphingomonas* spp. as well as the role of *Sphingomonadaceae* in the pathogenesis of donkey endometritis.

In the current study, endometritis of jennies was diagnosed based on clinical signs and laboratory test results including endometrial cytology and culture. All enrolled donkey jennies with endometritis had clinical signs of vaginal discharge, ultrasonographically detectable uterine fluid, and a history of infertility; therefore, clinical endometritis was the presumed diagnosis (Amos et al., 2014). Endometrial swab samples were further collected for cytology and bacterial culture in this study to confirm the diagnosis (Riddle et al., 2007). Uterine swabs (de Amorim et al., 2016), low-volume lavage (Frontoso et al., 2008), and cytobrush (LeBlanc et al., 2007) have been used for uterine sample collection. Although uterine swabs were less sensitive to cytologic evaluation (Katila, 2016) and might cause false negatives in uterine culture results (Cocchia et al., 2012), it is a quick and relatively easy method to perform (Nielsen et al., 2010). Swabs have been used to sample the uterine and vaginal microflora for high-throughput sequencing of metritis in dairy cows (Miranda-CasoLuengo et al., 2019). Hence, in the present study, double-guarded endometrial and vaginal swab samples were used for microbiome analysis. The microbiota samples were collected first to minimize the contamination of repetitively entering the reproductive tract. All jennies with endometritis were positive for both endometrial cytology assessment and bacterial culture growth, which indicate the relative validity of the swab samples in this study. The cytology and bacterial culture samples for healthy jennies were negative for endometritis, indicating the quality of samples was not impacted by repetitive entry into the uterus. There is also evidence that if sampling from a mare’s uterus was carried out using a double-guarded swab protected by a sterile obstetrical sleeve, the contamination is considered minimal and not significantly different from sampling by a low-volume lavage technique (Christoffersen et al., 2015). Another study showed that the uterine microbiomes detected in healthy mares did not reveal a significant difference between different sampling techniques including double-guarded swab, tissue biopsy, and low-volume lavage (Heil et al., 2018). Therefore, the swab samples of healthy jennies would be appropriate to detect the normal endometrial microbiome of donkey jennies in the current study. Negative control samples yielded a minimal amount of target DNA products, which ruled out the contamination impact from the sampling supply on the microbiota result in the current study. However, the number of negative controls was small, which was one of the limitations of this study.

## Conclusion

In this study, we report that the composition of vaginal and endometrial microbiomes in donkey jennies were unique, and endometritis might impact both endometrial and vaginal microbiota in donkey jennies. The significantly increased relative abundance of anaerobic bacterial taxa including *Ruminococcaceae* and *Lachnospiraceae* in endometrial and vaginal samples of endometritis jennies could be associated with the pathogenesis of donkey endometritis. The presence of a significantly higher abundance of *Sphingomonas* spp. in endometrial samples of endometritis jennies might be from the uncleared semen after breeding and could be linked to a risk of endometritis. Further research is warranted to investigate the association between these findings and pathogenesis of donkey endometritis.

## Data availability statement

The datasets presented in this study can be found in online repositories. The names of the repository/repositories and accession number(s) can be found at: https://www.ncbi.nlm.nih.gov/, PRJNA788693.

## Ethics statement

The animal study was reviewed and approved by the Animal Care and Use Committee of the China Agricultural University. Written informed consent was obtained from the owners for the participation of their animals in this study.

## Author contributions

SZ, JL, and GH conceived the study and reviewed and edited the manuscript. SZ, YZa, ZY, RW, and JM participated in animal acquisition and sample collection. YZu, ZY, RW, and ZW conducted data analysis. ZY, RW, and ZW contributed to the visualization. JL and YZu contributed to the writing of the original draft. All authors contributed to the article and approved the submitted version.

## Funding

This work was supported by the Introduction of Talent Research Start-up Fund from China Agricultural University [Grant number 31051017].

## Conflict of interest

The authors declare that the research was conducted in the absence of any commercial or financial relationships that could be construed as a potential conflict of interest.

## Publisher’s note

All claims expressed in this article are solely those of the authors and do not necessarily represent those of their affiliated organizations, or those of the publisher, the editors and the reviewers. Any product that may be evaluated in this article, or claim that may be made by its manufacturer, is not guaranteed or endorsed by the publisher.

## Supplementary material

SUPPLEMENTARY FIGURE 1 Rarefaction curves of endometrial and vaginal samples for microbiota analysis of all donkey jennies. **(A)** Rarefaction curves of endometrial samples of all jennies. **(B)** Rarefaction curves of vaginal samples of all jennies. Blue representing samples from healthy jennies and orange representing samples from endometritis jennies.

SUPPLEMENTARY FIGURE 2 Standard curve of 16S rRNA (V3-V4) clone.
