# Supplementary figures and images for: Endometrial and vaginal microbiome in donkeys with and without clinical endometritis
Source: Front Microbiol. 2022 Aug 1;13:884574. doi: 10.3389/fmicb.2022.884574 (PMC9376452; doi:10.3389/fmicb.2022.884574)

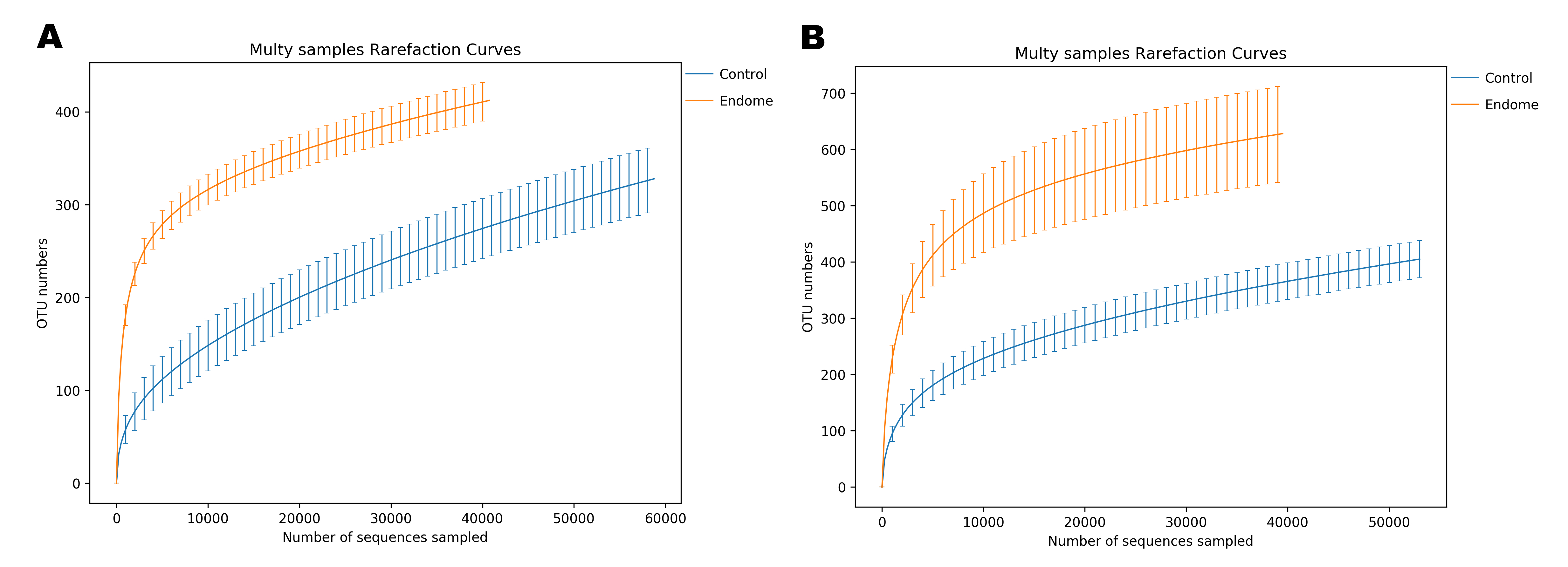

Supplement: SUPPLEMENTARY FIGURE 1 — Rarefaction curves of endometrial and vaginal samples for microbiota analysis of all donkey jennies. (A) Rarefaction curves of endometrial samples of all jennies. (B) Rarefaction curves of vaginal samples of all jennies. Blue representing samples from healthy jennies and orange representing samples from endometritis jennies. [file Image_1.JPEG]

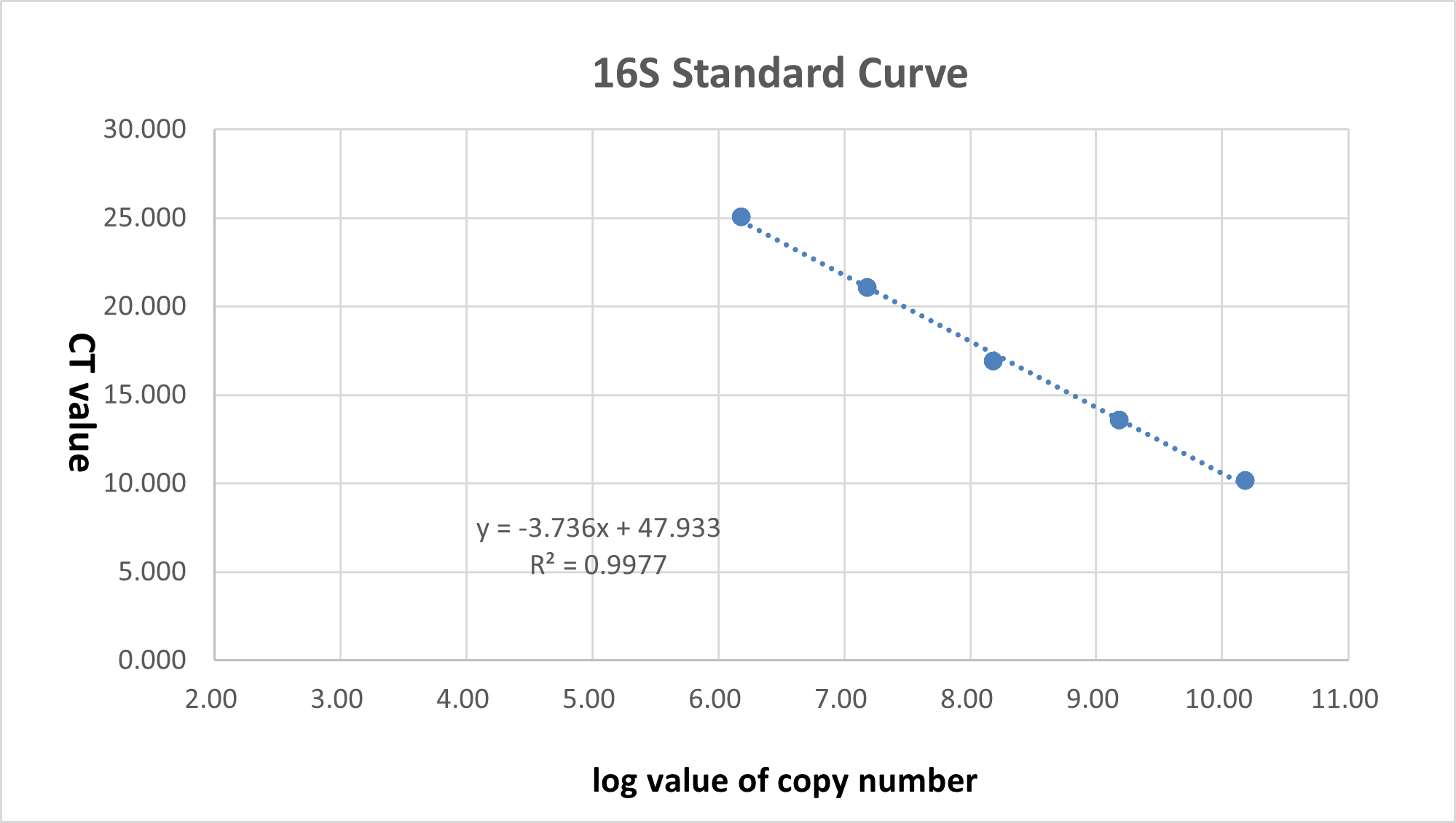

Supplement: SUPPLEMENTARY FIGURE 2 — Standard curve of 16S rRNA (V3-V4) clone. [file Image_2.PNG]
