# Supplementary material for: Endometrial and vaginal microbiome in donkeys with and without clinical endometritis
Source: Front Microbiol. 2022 Aug 1;13:884574. doi: 10.3389/fmicb.2022.884574 (PMC9376452; doi:10.3389/fmicb.2022.884574)
Supplement: Supplementary file 3 [file Data_Sheet_1.docx]

Supplementary table 1. Latest insemination record of donkey jennies in group E.

| ID of donkey jennies in group E (endometritis) | Age (year) | Last insemination date | Sampling date |
| --- | --- | --- | --- |
| 0115 | 6 | 9/04/2020 | 9/8/2020 |
| 0250 | 8 | 9/12/2020 | 9/17/2020 |
| 1057 | 5 | 9/15/2020 | 9/20/2020 |
| 1296 | 5 | 9/20/2020 | 9/25/2020 |
| 1489 | 4 | 10/13/2020 | 10/19/2020 |
| 1587 | 6 | 10/15/2020 | 10/19/2020 |
| 1781 | 7 | 10/20/2020 | 10/26/2020 |
| 2712 | 7 | 10/24/2020 | 10/29/2020 |
| 2794 | 6 | 11/01/2020 | 11/05/2020 |
| 2918 | 8 | 11/09/2020 | 11/14/2020 |

Supplementary table 2. The insemination history of donkey jennies in group C.

| ID of donkey jennies in group E (endometritis) | Age (year) | Insemination history |
| --- | --- | --- |
| 000 | 5 | 8/22/2019 (pregnant) |
| 0705 | 6 | 4/14/2018 (pregnant), 7/25/2019 (pregnant) |
| 1079 | 5 | 9/03/2019 (pregnant) |
| 1638 | 5 | 8/21/2019 (pregnant) |
| 1650 | 6 | 6/21/2018 (pregnant), 7/25/2019 (pregnant) |
| 180 | 5 | 5/30/2018 (pregnant), 8/05/2019 (pregnant) |
| 1888 | 4 | 9/06/2019 (pregnant) |
| 1914 | 7 | 6/22/2017 (pregnant), 8/14/2018 (pregnant), 8/16/2019 (pregnant) |
| 1917 | 7 | 4/04/2017 (pregnant), 6/19/2018 (pregnant), 8/28/2019 (pregnant) |
| 2649 | 5 | 8/07/2019 (pregnant) |
| 2815 | 5 | 8/16/2019 (pregnant) |
| 314 | 6 | 4/25/2017 (pregnant), 5/27/2018 (pregnant), 8/01/2019 (pregnant) |

Supplementary table 3. DNA concentration and read counts of all samples

| No. | Sample ID | Concentration(ng/μL) | Volume(μL) | Total DNA(μg) | Clean reads |
| --- | --- | --- | --- | --- | --- |
| Vaginal samples from group C | | | | | |
| 1 | Y2649 | 16.57 | 10 | 0.17 | 79,787 |
| 2 | Y1638 | 2.11 | 10 | 0.02 | 79,975 |
| 3 | Y1914 | 40 | 10 | 0.40 | 79,846 |
| 4 | Y1888 | 0.73 | 10 | 0.01 | 79,873 |
| 5 | Y0705 | 16.32 | 10 | 0.16 | 79,709 |
| 6 | Y000 | 15.33 | 10 | 0.15 | 79,575 |
| 7 | Y1650 | 18.54 | 10 | 0.19 | 79,799 |
| 8 | Y108 | 14.58 | 10 | 0.15 | 79,329 |
| 9 | Y1079 | 93.31 | 10 | 0.93 | 79,748 |
| 10 | Y1917 | 6.99 | 10 | 0.07 | 79,182 |
| 11 | Y314 | 33.85 | 10 | 0.34 | 79,657 |
| 12 | Y2815 | 2.3 | 10 | 0.02 | 79,833 |
| Endometria samples from group C | | | | | |
| 1 | Z2649 | 6.34 | 10 | 0.06 | 79,827 |
| 2 | Z1638 | 17.5 | 10 | 0.18 | 79,627 |
| 3 | Z1914 | 45.52 | 10 | 0.46 | 79,467 |
| 4 | Z1888 | 7.81 | 10 | 0.08 | 79,701 |
| 5 | Z0705 | 41.41 | 10 | 0.41 | 79.769 |
| 6 | Z000 | 8.28 | 10 | 0.08 | 79,754 |
| 7 | Z1650 | 14.76 | 10 | 0.15 | 79,741 |
| 8 | Z180 | 5.79 | 10 | 0.06 | 79,715 |
| 9 | Z1079 | 121.28 | 10 | 1.21 | 79.950 |
| 10 | Z1917 | 0.5 | 10 | 0.01 | 79,470 |
| 11 | Z314 | 24.64 | 10 | 0.25 | 80,028 |
| 12 | Z2815 | 39.91 | 10 | 0.40 | 79,350 |
| Vaginal samples from group E | | | | | |
| 1 | YN1489 | 6.99 | 10 | 0.07 | 79,591 |
| 2 | YN1781 | 2.26 | 10 | 0.02 | 79,717 |
| 3 | YN2794 | 42.06 | 10 | 0.42 | 79,728 |
| 4 | YN2712 | 5 | 10 | 0.05 | 80,005 |
| 5 | YN1296 | 2.03 | 10 | 0.02 | 79,659 |
| 6 | YN0250 | 3.55 | 10 | 0.04 | 80,082 |
| 7 | YN1587 | 0.65 | 10 | 0.01 | 79,769 |
| 8 | YN1057 | 1.44 | 10 | 0.01 | 79,433 |
| 9 | YN0115 | 12.3 | 10 | 0.12 | 79,655 |
| 10 | YN2918 | 4.66 | 10 | 0.05 | 79,884 |
| Endometria samples from group E | | | | | |
| 1 | ZN1489 | 54.43 | 10 | 0.54 | 61,501 |
| 2 | ZN1781 | 118.2 | 10 | 1.18 | 79,531 |
| 3 | ZN2794 | 38.1 | 10 | 0.38 | 79,840 |
| 4 | ZN2712 | 13.42 | 10 | 0.13 | 79,672 |
| 5 | ZN1296 | 226.83 | 10 | 2.27 | 79,496 |
| 6 | ZN0250 | 6.53 | 10 | 0.07 | 79,632 |
| 7 | ZN1587 | 14.39 | 10 | 0.14 | 79,824 |
| 8 | ZN1057 | 184.16 | 10 | 1.84 | 79,867 |
| 9 | ZN0115 | 70.72 | 10 | 0.71 | 79,989 |
| 10 | ZN2918 | 156.49 | 10 | 1.56 | 79,779 |

Supplementary table 4. Average of copy number of all samples and negative controls

| Name of genes | No. | Sample ID | Average of copy number (1μL template DNA) |
| --- | --- | --- | --- |
| 16s rRNA (V3-V4) | Vaginal samples from group C | | |
|  | 1 | Y2649 | 482719635 |
|  | 2 | Y1638 | 2982564 |
|  | 3 | Y1914 | 8771661 |
|  | 4 | Y1888 | 82698 |
|  | 5 | Y0705 | 106007119 |
|  | 6 | Y000 | 181088188 |
|  | 7 | Y1650 | 14545339 |
|  | 8 | Y108 | 29855797 |
|  | 9 | Y1079 | 109613182 |
|  | 10 | Y1917 | 15663640 |
|  | 11 | Y314 | 1702406016 |
|  | 12 | Y2815 | 844419 |
|  | Endometria samples from group C | | |
|  | 1 | Z2649 | 1036922254 |
|  | 2 | Z1638 | 224056021 |
|  | 3 | Z1914 | 226770881 |
|  | 4 | Z1888 | 4235781 |
|  | 5 | Z0705 | 379657632 |
|  | 6 | Z000 | 63729998 |
|  | 7 | Z1650 | 50025315 |
|  | 8 | Z180 | 1006807 |
|  | 9 | Z1079 | 1906595791 |
|  | 10 | Z1917 | 85063434 |
|  | 11 | Z314 | 343205872 |
|  | 12 | Z2815 | 418982147 |
|  | Vaginal samples from group E | | |
|  | 1 | YN1489 | 126120878 |
|  | 2 | YN1781 | 451879 |
|  | 3 | YN2794 | 753272 |
|  | 4 | YN2712 | 87649 |
|  | 5 | YN1296 | 1599288 |
|  | 6 | YN0250 | 16320 |
|  | 7 | YN1587 | 149236 |
|  | 8 | YN1057 | 7589355 |
|  | 9 | YN0115 | 318374 |
|  | 10 | YN2918 | 91622197 |
|  | Endometria samples from group E | | |
|  | 1 | ZN1489 | 159599 |
|  | 2 | ZN1781 | 113958 |
|  | 3 | ZN2794 | 280572 |
|  | 4 | ZN2712 | 15589 |
|  | 5 | ZN1296 | 136319 |
|  | 6 | ZN0250 | 29526 |
|  | 7 | ZN1587 | 155911 |
|  | 8 | ZN1057 | 57467 |
|  | 9 | ZN0115 | 83571 |
|  | 10 | ZN2918 | 11522444 |
|  | Negative control samples | | |
|  | 1 | Negative control 1 | 0 |
|  | 2 | Negative control 2 | 0 |
|  | 3 | Negative control 3 | 0 |
